# Design and methods of the NiCK study: neurocognitive assessment and magnetic resonance imaging analysis of children and young adults with chronic kidney disease

**DOI:** 10.1186/s12882-015-0061-1

**Published:** 2015-04-30

**Authors:** Erum A Hartung, Nina Laney, Ji Young Kim, Rebecca L Ruebner, John A Detre, Hua-Shan Liu, Christos Davatzikos, Guray Erus, Jimit J Doshi, Robert T Schultz, John D Herrington, Abbas F Jawad, Divya G Moodalbail, Ruben C Gur, Allison M Port, Jerilynn Radcliffe, Stephen R Hooper, Susan L Furth

**Affiliations:** Division of Nephrology, Children’s Hospital of Philadelphia, 34th and Civic Center Boulevard, Philadelphia, PA USA; Department of Pediatrics, Perelman School of Medicine at the University of Pennsylvania, Philadelphia, PA USA; Biostatistics Core, Clinical and Translational Research Center, Children’s Hospital of Philadelphia, Philadelphia, PA USA; Department of Neurology, Perelman School of Medicine at the University of Pennsylvania, Philadelphia, PA USA; Graduate Institute of Clinical Medicine and Imaging Research Center, College of Medicine, Taipei Medical University, Taipei, Taiwan; Department of Medical Imaging, Taipei Medical University Hospital, Taipei, Taiwan; Center for Biomedical Image Computing and Analytics, Department of Radiology, Perelman School of Medicine at the University of Pennsylvania, Philadelphia, PA USA; Center for Autism Research, Children’s Hospital of Philadelphia, Philadelphia, PA USA; Department of Psychiatry, Perelman School of Medicine at the University of Pennsylvania, Philadelphia, PA USA; Children’s Hospital of Philadelphia, Philadelphia, PA USA; Division of Pediatric Nephrology, Department of Pediatrics, Nemours/Alfred I. duPont Hospital for Children, Wilmington, DE USA; Brain and Behavior Laboratory, Department of Psychiatry, University of Pennsylvania, Philadelphia, PA USA; Division of Developmental and Behavioral Pediatrics, Children’s Hospital of Philadelphia, Philadelphia, PA USA; Department of Allied Health Sciences, University of North Carolina School of Medicine, Chapel Hill, NC USA; Department of Epidemiology, Perelman School of Medicine at the University of Pennsylvania, Philadelphia, PA USA

**Keywords:** Neurocognition, Neuropsychological, Chronic kidney disease, Hypertension, Cerebrovascular disease, Cardiovascular disease, Neuroimaging, Magnetic resonance imaging, Children, Adolescents, Adults

## Abstract

**Background:**

Chronic kidney disease is strongly linked to neurocognitive deficits in adults and children, but the pathophysiologic processes leading to these deficits remain poorly understood. The NiCK study (Neurocognitive Assessment and Magnetic Resonance Imaging Analysis of Children and Young Adults with Chronic Kidney Disease) seeks to address critical gaps in our understanding of the biological basis for neurologic abnormalities in chronic kidney disease. In this report, we describe the objectives, design, and methods of the NiCK study.

**Design/methods:**

The NiCK Study is a cross-sectional cohort study in which neurocognitive and neuroimaging phenotyping is performed in children and young adults, aged 8 to 25 years, with chronic kidney disease compared to healthy controls. Assessments include (1) comprehensive neurocognitive testing (using traditional and computerized methods); (2) detailed clinical phenotyping; and (3) multimodal magnetic resonance imaging (MRI) to assess brain structure (using T1-weighted MRI, T2-weighted MRI, and diffusion tensor imaging), functional connectivity (using functional MRI), and blood flow (using arterial spin labeled MRI). Primary analyses will examine group differences in neurocognitive testing and neuroimaging between subjects with chronic kidney disease and healthy controls. Mechanisms responsible for neurocognitive dysfunction resulting from kidney disease will be explored by examining associations between neurocognitive testing and regional changes in brain structure, functional connectivity, or blood flow. In addition, the neurologic impact of kidney disease comorbidities such as anemia and hypertension will be explored. We highlight aspects of our analytical approach that illustrate the challenges and opportunities posed by data of this scope.

**Discussion:**

The NiCK study provides a unique opportunity to address key questions about the biological basis of neurocognitive deficits in chronic kidney disease. Understanding these mechanisms could have great public health impact by guiding screening strategies, delivery of health information, and targeted treatment strategies for chronic kidney disease and its related comorbidities.

## Background

Chronic kidney disease (CKD) is strongly linked to neurocognitive dysfunction in adults and children [1-4], but the pathophysiologic processes leading to these deficits remain poorly understood. A number of studies have used neuroimaging to characterize the neuroanatomic changes in children and adults with CKD. A recent systematic review [5] of such studies identified three major types of abnormalities: (1) cerebral atrophy and cerebral density changes; (2) signs of cerebral vascular disease; and (3) regional blood flow changes. While the majority of these studies were performed in adults, the small number of pediatric studies highlighted some overlapping findings, including cerebral atrophy and periventricular white matter infarcts [6-10].

Although these studies have increased awareness of neuroanatomical abnormalities in CKD, a key question remains: What are the specific physiologic disturbances in CKD that lead to neurocognitive changes? Potential mechanisms include metabolic neuronal injury [11] and, perhaps more importantly, subclinical vascular disease [4], mediated through cardiovascular risk factors such as anemia, dyslipidemia, and hypertension. However, it remains unclear how clinical risk factors in CKD produce specific neurophysiologic changes, and how these changes translate to clinically relevant neurocognitive outcomes.

The Neurocognitive Assessment and Magnetic Resonance Imaging Analysis of Children and Young Adults with Chronic Kidney Disease (NiCK) Study was designed to address this critical gap in our understanding of the biological basis for neurologic abnormalities in CKD. With funding from the Pennsylvania Department of Health, this study uses a comprehensive interdisciplinary approach and leverages unique resources at the Children’s Hospital of Philadelphia and the University of Pennsylvania to combine comprehensive neurocognitive assessments and state-of-the-art multimodal magnetic resonance imaging (MRI) in children and young adults with CKD.

In this report, we describe the objectives, design, and methods of the NiCK study, and outline how the study provides the opportunity to address key questions in the field. We will then describe our approach to data analysis, focusing on the challenges and opportunities posed by the multitude of variables inherent in clinical, neurocognitive, and neuroimaging data of this scope. Finally, we will outline future directions for this rich dataset, including the potential for novel unbiased scientific discovery, and discuss the potential public health impact of this study.

### Objective

The overarching objectives of the NiCK study are (1) to determine how CKD and its associated comorbidities affect neurocognitive function, and (2) to understand the neurobiological basis for cognitive abnormalities in CKD. The NiCK study seeks to achieve these objectives using the following three-pronged approach:

1. Detailed neurocognitive description of children and young adults with CKD, along with concurrent age-matched healthy controls, using:
  - a comprehensive battery of traditional neurocognitive testing, and
  - a computerized neurocognitive battery
2. Detailed clinical description to explore the impact of clinical risk factors such as hypertension and anemia; and
3. Multimodal magnetic resonance imaging (MRI) to identify differences in brain structure, functional connectivity, and cerebral blood flow, using:
  - ***Structural MRI*** to explore the effects of CKD on brain structure
  - ***Resting state blood oxygenation level dependent functional MRI*** (BOLD fMRI) to evaluate connectivity within brain networks that modulate specific neurocognitive functions
  - ***Arterial spin labeled perfusion MRI*** to evaluate global and regional cerebral blood flow.

This multifaceted approach will allow us to integrate findings from neurocognitive testing, clinical phenotyping, and multimodal MRI to develop a unique multi-parametric neurocognitive phenotype for CKD. This innovative approach to assessment will provide new knowledge to further our understanding of mechanisms underlying neurological dysfunction in CKD.

## Methods/design

### Study design

The NiCK study consists of a cross-sectional study of 90 pediatric and young adult subjects, aged 8 – 25 years, with Stage 2 to 5 CKD (estimated glomerular filtration rate [eGFR] < 90 mL/min/1.73 m^2^, including dialysis and post-transplant), compared to healthy controls matched on age and socioeconomic status (using insurance status as a proxy).

The study was carried out in accordance with the Declaration of Helsinki and was approved by the Institutional Review Board of the Children’s Hospital of Philadelphia. Written informed consent was obtained from all participants or their parents/legal guardians for subjects under age 18 years.

### Setting

The NiCK study is performed at a large tertiary care children’s hospital in an urban setting (the Children’s Hospital of Philadelphia).

### Study participants

Table 1 shows the inclusion and exclusion criteria for NiCK study participants. Eligibility for CKD participants is based on eGFR using the bedside CKiD equation [12] for participants aged 8 to 18 years, and the Modification of Diet in Renal Disease (MDRD) study equation [13] for those over 18 years of age. CKD is defined as evidence of kidney dysfunction for more than six months.

**Table 1:** **Inclusion and exclusion criteria for the NiCK study**

**Inclusion criteria**	Age 8 – 25 years
	English is primary language for participant (most neurocognitive measures standardized only in English). Parent completing questionnaires must be proficient in English.
	CKD subjects: Stage 2–5 CKD (eGFR < 90 mL/min/1.73 m^2^), including dialysis and transplant patients
	Control subjects: Healthy siblings or individuals matched in age and insurance status
**Exclusion criteria**	Conditions that would prohibit MRI: certain types of body metal; claustrophobia
	Auditory impairment (that cannot be corrected by a hearing aid) that would significantly impede the valid collection of test measures
	History of traumatic brain injury or other significant medical or neurological abnormality affecting motor or higher cortical functioning (e.g. seizure disorder, genetic syndromes, systemic diseases that can affect the brain such as sickle cell disease, cerebral lupus, spina bifida, gestational age below 32 weeks, or perinatal injury)
	Profound developmental disability or sensory-motor difficulties that would preclude valid use of diagnostic instruments or scanning procedures.
	A severe DSM IV-TR Axis I disorder or other psychiatric symptoms that would interfere with the participant’s ability to participate in the study (e.g., active psychosis)
	Known drug or alcohol use within 24 hours of any assessment
	Pregnancy (because of potential risk of MRI to the unborn fetus).

CKD, chronic kidney disease; eGFR, estimated glomerular filtration rate (using the bedside CKiD equation [12] for subjects aged <18 years or the MDRD equation [13] for those ≥ 18 years); DSM IV-TR, Diagnostic and Statistical Manual of Mental Disorders, 4th Edition, Text Revision; MRI, magnetic resonance imaging.

The lower age limit of 8 years ensures improved compliance with imaging procedures. Since the selected neurocognitive measures are standardized largely in English, participants must have English as their primary language. To ensure that study results reflect the effects of kidney disease, patients with a number of comorbidities that independently affect brain function (e.g., seizure disorder) or the ability to complete test measures (profound developmental disabilities) are excluded from participating (see Table 1).

### Study procedures

The participant data collected include demographic information (age at visit, age at CKD diagnosis, gender, maternal education, insurance status, race), past medical history, family history, and current medications. Participants then undergo clinical, imaging, and neurocognitive assessments as described below.

### Clinical evaluations

Participants’ blood pressure (BP), heart rate, respirations, height, weight, and body mass index are measured. Laboratory data collected includes complete blood count, comprehensive metabolic panel, calcium, phosphate, cystatin C, lipid panel, and urine studies for total protein, albumin, and creatinine. Urine pregnancy testing is performed in post-pubertal girls prior to MRI scanning. All participants undergo 24-hour ambulatory blood pressure monitoring (ABPM).

### Neurocognitive assessments

#### Traditional neurocognitive battery

A battery of age-specific neurocognitive assessments (Table 2) is performed at the baseline visit to measure various aspects of attention/executive functioning via laboratory and parent/participant ratings. In addition, age-specific depression indices and a visual analog anxiety scale are administered. To address the potential effects of fatigue and attention-loss on test performance, the tests are administered in a counterbalanced format across each study participant. A behavior coding mechanism is used to provide examiner perception of the reliability and validity of the test data collected.

**Table 2:** **Traditional neurocognitive and affective measurements**

**Domain**	**Test name**	**Age group for test in this study**
Intelligence	Wechsler Abbreviated Scales of Intelligence (WASI)	All
Attention Regulation	Conners’Continuous Performance Test II (CPT-II)	All
	Delis-Kaplan Executive Function System Tower Subtest (D-KEFS Tower)	All
Working Memory	Wechsler Intelligence Scale for Children Fourth Edition Integrated (WISC-IV-I) Digit Span Task	16 years or younger
	Wechsler Intelligence Scale for Children Fourth Edition Integrated (WISC-IV-I) Spatial Span Task	16 years or younger
	Wechsler Memory Scale Third Edition (WMS-III) Digit Span Task	17 years or older
	Wechsler Memory Scale Third Edition (WMS-III) Spatial Span Task	17 years or older
Executive Function Behavior	Behavior Rating Inventory of Executive Function (BRIEF)	17 years or younger
	BRIEF – Adult Version (BRIEF-A)	18 years or older
Depression	Children’s Depression Inventory II Short (CDI-2 Short)	17 years or younger
	Beck Depression Inventory II (BDI-II)	18 years or older
Anxiety	Visual Analog Anxiety Scale	All

#### Computerized neurocognitive battery

In addition to the traditional neurocognitive assessments outlined in Table 2, participants also undergo a computerized neurocognitive battery (CNB) developed at the University of Pennsylvania [14]. The Penn CNB includes 14 tests assessing five neurobehavioral functions (Table 3): executive control, episodic memory, complex cognition, social cognition, and praxis speed. The Penn CNB assesses both accuracy (proportion of correct responses) and speed (response time for correct responses), with the latter variable providing an additional area of exploration. The Penn CNB requires approximately one hour to administer. Age-specific norms for the Penn CNB are available based on data from a large cohort (n ≈ 1800) of typically developing children and young adults in the Philadelphia Neurodevelopmental Cohort [15].

**Table 3:** **Domains and tests in the Penn computerized neurocognitive battery**

**Neurobehavioral function**	**Domain**	**Test name**	**Label**
Executive Control	Abstraction/mental flexibility	Penn Conditional Exclusion Test	PCET
	Attention	Penn Continuous Performance Test	PCPT
	Working memory	Short Letter N-Back Test	SLNB
Episodic Memory	Verbal Memory	Penn Word Memory	CPW
	Facial Memory	Penn Face Memory	CPF
	Spatial Memory	Visual Object Learning Test	VOLT
Complex Cognition	Verbal Reasoning	Penn Verbal Reasoning Test	PVRT
	Nonverbal Reasoning	Penn Matrix Reasoning Test	PMAT
	Spatial Processing	Penn Line Orientation Test	PLOT
Social Cognition	Emotion Identification	Penn Emotion Identification Test	ER40
	Emotion Differentiation	Penn Emotion Differentiation Test	MEDF
	Age Differentiation	Penn Age Differentiation Test	ADT
Praxis Speed	Sensorimotor Speed	Motor Praxis	MPRAXIS
	Motor Speed	Finger Tapping Test	CTAP

### Imaging measurements

After appropriate MRI safety screening, participants undergo non-sedated, non-contrast MRI of the brain, acquired on a Siemens Verio 3 T scanner equipped with a 32-channel head coil. The FDA- and manufacturer-approved sequences used are shown in Table 4. The Physiologic Monitoring Unit (PMU) of the Siemens Verio is used to collect pulse oxygenation data during the scan. All images are read by board certified pediatric neuroradiologists, and all clinically significant incidental findings are communicated to the participants.

**Table 4:** **Magnetic resonance imaging (MRI) Sequences performed in the NiCK study**

**Name**	**Sequence parameters**
3D T1 MPRAGE	TR = 1.79 s, TE = 3.06 ms, TI = 1.050 s, FoV = 250 × 250 mm^2^, flip angle = 10°, voxel size = 1 × 1 × 1 mm^3^
pCASL 2D GE EPI	TR = 4 s, TE = 12 ms, FoV 220 × 220 mm^2^, flip angle = 90°, voxel size = 3.4 × 3.4 × 5.0 mm^3^, labeling duration 1.5 s, postlabeling delay 1.2 s, 40 label/control pairs
T2 FLAIR	TR = 9 s, TE = 76 ms, TI = 2.5 s, FoV = 220 × 220 mm^2^, flip angle = 146°, voxel size = 0.9 × 0.9 × 2 mm^3^
T1 FLASH	TR = 300 ms, TE = 2.46 ms, FoV = 225 × 225 mm^2^, flip angle = 60°, voxel size = 0.9 × 0.9 × 3 mm^3^
GRE Field Mapping	TR = 499 ms, TE1 = 5.19 ms, TE2 = 7.65 ms, FoV = 192 × 192 mm^2^, flip angle = 60°, voxel size = 3 × 3 × 3 mm^3^
Resting fMRI/GE EPI	TR = 3 s, TE 30 ms, FoV = 192 × 192 mm^2^, flip angle = 90°, voxel size = 3 × 3 × 3 mm^3^ isotropic, 102 volumes
DTI SE EPI	TR = 11 s, TE = 76.4 ms, b = 0 and 1000 s/mm^2^, 30 directions, FoV = 256 × 256 mm^2^, voxel size = 2 × 2 × 2 mm^3^
Proton Density/T2 MRI	TR = 6.22 s, TE1= 14 ms, TE2 = 98 ms, FoV = 256 × 256 mm^2^, flip angle = 150°, voxel size = 1 × 1 × 2 mm^3^

MPRAGE, magnetization prepared rapid acquisition gradient echo; pCASL, pseudocontinuous arterial spin labeling; GE EPI, gradient-echo echo-planar imaging; FLAIR, fluid-attenuated inversion recovery; FLASH, fast low angle shot; GRE, gradient recalled echo; fMRI, functional MRI; DTI, diffusion tensor imaging; PD, proton density; T1, T2, weighting of applied MRI sequence; TR, repetition time; TE, echo time; TI, inversion time; FoV, field of view.

#### Structural MRI (sMRI)

An automated processing pipeline consisting of extensively validated methods is applied for processing sMRI images. The processing pipeline includes: extraction of the brain parenchymal tissue using multi-atlas skull-stripping [16]; inhomogeneity correction and tissue segmentation into gray matter (GM), white matter (WM) and cerebrospinal fluid (CSF) [17]; formation of regional volumetric maps, called RAVENS (regional analysis of volumes examined in normalized space) maps [18] using a deformable atlas registration method [19] to enable comparative analysis of tissue volumes in the common template space; segmentation of white matter lesions using a multi-modal supervised learning method [20]; and segmentation into a set of expert-defined anatomic regions of interest (ROIs) using a recently developed multi-atlas label fusion method [21]. The segmentation included 148 ROIs, including 98 cortical regions, white matter regions partitioned into brain lobes, as well as important deep structures such as hippocampus, thalamus and amygdala. The ROIs are organized within a hierarchical structure such that quantitative imaging measurements from normal and abnormal (lesion) tissues could be calculated from different image modalities for both individual ROIs and larger anatomical regions.

By summarizing the very high dimensional image data with much fewer variables, calculated volumetric measurements allow us to perform quantitative analyses of regional brain volumes and fractional anisotropy, and to evaluate the extent of brain atrophy and cerebrovascular disease such as infarcts and leukoareosis.

#### Blood oxygenation level dependent functional MRI (BOLD fMRI)

BOLD fMRI is used to evaluate connectivity within specific cognitive networks in the absence of external stimuli (resting state BOLD fMRI). Following standard registration procedures (implemented via the program FIRST) [22], fMRI data are registered to the standard space of the Montreal Neurological Institute (MNI) template. Data analysis focuses primarily on seed-based multiple regression to identify differences in attention networks (for example, the Default Mode Network, or DMN [23]). Multiple regression follows the procedures outlined by Satterthwaite et al. [24], including a variety of nuisance parameters including global fMRI signal, white matter and CSF signal, and head motion. The resulting correlation (r) maps are converted to z-statistic maps (via Fisher r-to-z transformation), which are then used as descriptive statistics for group analyses and per-voxel correlations with clinical and neuropsychological variables.

#### Arterial spin labeled (ASL) MRI

ASL MRI uses spin-labeled blood water as an endogenous tracer to measure regional cerebral blood flow (CBF). This study acquired pseudocontinuous ASL [25] sampled with 2-dimensional echoplanar imaging (EPI). ASL images are processed using a perfusion data processing toolbox, ASLtbx [26]. The raw EPI images are first motion-corrected, then pairwise subtraction images are generated from images acquired with and without arterial labeling. The averaged difference images are used to calculate CBF (in mL/100 g/min) as described by Wang et al. [27]. Each subject’s data are then segmented into GM, WM, whole-brain CBF maps that are used for group comparisons or correlations with clinical or neurocognitive indices. The ASL images are also normalized to the standard space of the MNI brain template for voxel-based analyses.

### Study size

A sample size of 90 CKD subjects and 90 healthy controls was calculated based on the assumption that the prevalence of MRI abnormalities in CKD subjects will be 12.5% and will be 1% in normal controls. This sample size provides 80% power to detect such a difference with 95% confidence.

### Key questions addressed by this study

The comprehensive approach employed in the NiCK study provides the opportunity to address some key issues and challenges in the field.

#### How do cardiovascular risk factors contribute to neurocognitive dysfunction in CKD, and does CKD affect neurocognition independent of its vascular effects?

Vascular disease is a well-known independent risk factor for cognitive impairment and dementia in adults [28]. Adults with CKD are at increased risk for stroke, silent brain infarcts, white matter lesions, and dementia, presumably due to cerebral small-vessel disease [4]. However, the extent to which CKD affects neurocognition independently of its vascular effects is unclear [29]. Discerning a CKD-specific effect is difficult in adults due to the high prevalence of pre-existing cardiovascular disease. In contrast, children with CKD generally have fewer pre-existing cardiovascular comorbidities. Studying children and young adults with CKD therefore provides the opportunity to identify neurologic changes that may be specific to CKD.

By comparing neurocognitive test results with MRI data, we will be able to evaluate whether specific deficits correlate with changes in structure, connectivity, or CBF in particular regions of the brain. For example, executive function is a domain known to be affected in children with CKD [3]. We would therefore hypothesize that children with deficits in executive function may have abnormalities in the prefrontal cortex and frontotemporal regions, such as white matter changes or volume loss on sMRI, or regional CBF changes on ASL MRI. The impact of level of kidney function on specific imaging parameters can be evaluated. In addition, the effect of cardiovascular risk factors can be analyzed, such as hypertension, anemia, dyslipidemia, and calcium-phosphate product. Examining these changes in children and young adults may help to elucidate targets for early intervention.

#### By what mechanisms do risk factors such as hypertension contribute to neurocognitive deficits in CKD?

Hypertension has been associated with neurocognitive dysfunction in adults [30-32] and children [33,34] with and without CKD. Possible mechanisms include vascular remodeling, altered cerebrovascular reactivity, or direct neuronal effects [35-37], but there are few studies that directly explore the neurophysiologic impact of hypertension [30-32]. The NiCK study provides a unique opportunity to address this gap in knowledge by combining detailed clinical phenotyping including casual BP measurements and ABPM, with comprehensive neurocognitive testing and multimodal MRI. This strategy allows us to identify specific neurocognitive deficits caused by hypertension, and directly correlate them with global or regional brain abnormalities such as changes in CBF on ASL MRI, or changes in functional connectivity on resting state BOLD fMRI.

#### How does CKD affect neurocognitive function and structure at different stages of development?

Brain maturation involves a complex series of structural and functional changes throughout childhood, adolescence, and young adulthood [38]. By studying cognitive function and brain structure in a cross-sectional sample of participants aged 8–25 years with and without CKD, the NiCK study will allow us to explore how CKD and its related comorbidities affect neurodevelopment at different ages. This may allow us to identify targets for early screening or therapeutic intervention.

### Approaches to data analyses and challenges

The data collected in the NiCK study provides great opportunities to test hypotheses regarding differences in brain structure and function between individuals with CKD and healthy controls. However, the heterogeneous nature of CKD participants in this study presents both challenges and opportunities. Their wide range of kidney function (from dialysis-dependent up to eGFR 90 mL/min/1.73 m^2^) allows us to explore neurologic changes across the continuum of disease severity. However, this diversity also introduces a greater number of covariates that must be accounted for in our analyses.

Analysis of the large number of clinical and imaging variables also presents significant challenges, particularly in developing strategies to handle multiple comparisons. For example, each neurocognitive testing method involves numerous individual tasks and scores; ABPM produces dozens of summary measures; and each MRI image consists of millions of voxels. The analysis plan for this study therefore includes a number of strategies for data reduction and for comparisons of measures across various modalities.

### Clinical variables

#### Kidney disease severity

**Figure 1:**
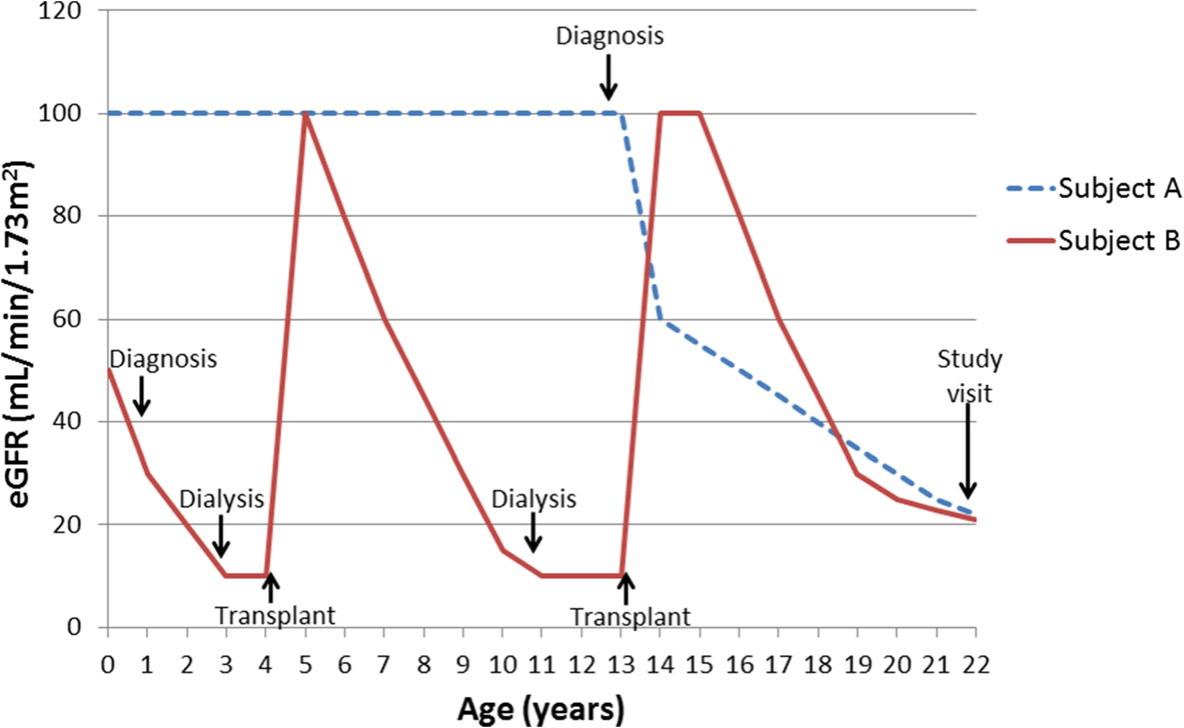
Illustration of the value of the severity score.Trajectory of estimated glomerular filtration rate (eGFR) of two hypothetical subjects of the same age with the same eGFR at the time of the study visit. Despite the similarity in their current kidney function, subject B’s course may have inflicted greater neurocognitive insult compared to that of subject A. This difference in clinical course is reflected in their Severity Scores (5 for subject B vs. 2 for subject A – see Table 5), but would be missed if we only considered current eGFR.

To determine the effect of CKD on neurocognitive function and neuroimaging parameters, our primary analytic approach will be group comparisons between CKD and control subjects. However, it is important to recognize that the wide range of kidney function in the CKD group could obscure effects of more severe kidney disease on neurocognitive function; consequently, other measures of CKD severity need to be considered. Further, while eGFR would seem to be a logical marker of kidney disease severity, this study includes post-transplant subjects and current eGFR may not account for other elements of disease trajectory that could affect neurodevelopment. To try to overcome this limitation, we devised a Severity Score (Table 5) to use in our analyses. Based on an additive cumulative risk model, this score includes additional indicators of past disease activity – whether the subject has ever required dialysis or transplant, and whether renal replacement therapy was required at age ≤ 5 years, a critical developmental window. These factors are combined with a score for eGFR groupings, to result in a final Severity Score that ranges from 0 (least severe) to 6 (most severe). The Severity Score approach is simple, and by virtue of being based on objectively determined criteria, it is not subject to inter-rater variability. The additional information gained by the Severity Score is illustrated in Figure 1, which represents the clinical course of two hypothetical subjects of the same age with the same eGFR at the time of the study visit. Despite the similarity in their current kidney function, it is clear that subject B’s trajectory has the potential to inflict greater neurological and neurocognitive insult compared to that of subject A – this is reflected in a higher Severity Score (5 for subject B vs. 2 for subject A). Use of the Severity Score in our analyses therefore allows us to include these additional factors that could affect neurodevelopmental outcome, and contributes to data reduction.

**Table 5:** **Severity score system**

**Clinical characteristics**	**Points**
Past disease activity score	
Has the subject ever received dialysis?	No = 0, Yes = 1
Has the subject ever received a kidney transplant?	No = 0, Yes = 1
Did the subject receive dialysis or transplant at age ≤ 5 years?	No = 0, Yes = 1
Current estimated glomerular filtration rate (eGFR, ml/min/1.73m^2^) score	
eGFR > 60	0
30 < eGFR ≤ 60	1
15 < eGFR ≤ 30	2
eGFR ≤ 15	3
**Severity Score (past disease activity score + current eGFR score)**	**0 to 6**

eGFR, estimated glomerular filtration rate (using the bedside CKiD equation [12] for subjects aged <18 years or the MDRD equation [13] for those ≥ 18 years).

#### Ambulatory blood pressure monitoring

There are a large number of blood pressure (BP) variables generated by 24-hour ABPM. These include measures for systolic and diastolic BP (SBP and DBP) and heart rate (HR), and can be broken down into waking hours, sleeping hours, and the total 24 hours. Potential variables include mean BP, mean BP index (BP normalized for age, height, and gender), BP load (proportion of readings exceeding the 95th percentile for age, height, and gender), nocturnal BP dipping (percent decrease in mean BP from waking to sleeping), hyperbaric index (area enclosed by the line of ambulatory BP readings above and the 95th percentile limit line for BP readings below), and BP and HR variability (coefficient of variation of BP readings and HR). Many of these variables are highly correlated with each other (e.g. mean BP and BP load); however, a growing number of studies show that certain ABPM variables, for example abnormal nocturnal dipping or increased BP variability, are independent predictors of adverse cardiovascular and neurocognitive outcomes. Our approach in selecting which ABPM variables to focus our analyses on has been two-pronged: (1) review of the literature to create an *a priori* list of variables expected to have the greatest clinical significance, and (2) assessment for correlation between ABPM variables – the variables chosen for our primary analyses were those that were not highly correlated with each other, and thus potentially most informative about different aspects of BP physiology.

### Neurocognitive assessments

This study includes comprehensive neurocognitive phenotyping in youth with CKD. Although traditional neurocognitive batteries (TNB) have been used extensively in individuals with CKD, this is the first study to utilize a computerized testing (the Penn CNB) in this population. Both the TNB and CNB provide comprehensive assessments of multiple neurocognitive domains. However, since the exact tests differ between the two batteries, one challenge we encounter is aligning specific tasks in each battery with neurocognitive functions tested. Table 6 demonstrates the framework used in this study for mapping tests within the TNB and CNB to various neurocognitive domains. As the table illustrates, some neurocognitive domains are tested by multiple components of the TNB and CNB. At the same time, some tests can span several neurocognitive domains (for example, the Penn Verbal Reasoning Test [PVRT] assesses both language and planned problem solving). Since performance across various tests and domains can be highly correlated, approaches such as factor analysis and principal component analysis are required in our analyses.

**Table 6:** **Framework for mapping tests/tasks in the traditional and computerized neurocognitive batteries to specific neurocognitive domains**

**Domain**	**Traditional neurocognitive battery (TNB) tests**	**Computerized neurocognitive battery (CNB) tests**
Language	WASI Vocabulary Subtest T-Score	Language Reasoning (PVRT)
	WASI Similarities Subtest T-Score	
Attention	WMS-III Digit Span Forward Scaled Score/WISC-IV-I Digit Span Forward Scaled Score	Attention (PCPT)
	WMS-III Spatial Span Forward Scaled Score/ WISC-IV-I Spatial Span Forward Scaled Score	
	CPT-II Omissions T-Score	
	CPT-II Variability T-Score	
	CPT-II Detectability T-Score	
	CPT-II Response Style T-Score	
Inhibitory Control	CPT-II Commissions T-Score	False Positive (i.e. incorrect) scores from:
		Working Memory (SLNB)
	CPT-II Hit RT T-Score	Attention (PCPT)
Planned problem solving	WASI Matrix Reasoning Subtest T Score	Nonverbal Reasoning (PMAT)
	D-KEFS Total Achievement Scaled Score	Language Reasoning (PVRT)
	D-KEFS Mean First-Move Time Scaled Score	
Set Shifting	D-KEFS Move Accuracy Ratio Scaled Score	Abstraction/Mental Flexibility (PCET)
Visual Spatial	WASI Block Design Subtest T Score	Nonverbal Reasoning (PMAT)
	WASI Matrix Reasoning Subtest T Score	Spatial Processing (PLOT)
Verbal Working Memory	WMS-III Digit Span Backward Scaled Score/WISC-IV-I Digit Span Backward Scaled Score	Working Memory (SLNB)
Visual Working Memory	WMS-III Spatial Span Backward Scaled Score/WISC-IV-I Spatial Span Backward Scaled Score	Working Memory (SLNB)
Verbal Memory	WMS-III Digit Span Forward Scaled Score/WISC-IV-I Digit Span Forward Scaled Score	Verbal Memory (CPW)
Visual Memory	WMS-III Spatial Span Forward Scaled Score/ WISC-IV-I Spatial Span Forward Scaled Score	Facial Memory (CPF)
		Spatial Memory (VOLT)
Ratings of Executive Function	Global Executive Composite	Abstraction/Mental Flexibility (PCET)
Social Cognition	None	Emotion Identification (ER40)
		Emotion Differentiation (MEDF)
		Age Differentiation (ADT)
Motor Speed	CPT-II Hit RT T-Score	Sensorimotor Speed (MPRAXIS)
		Motor Speed (CTAP)

Abbreviations for TNB tests defined in Table 2, and for CNB tests in Table 3.

Interpretation of performance across the TNB and CNB presents another challenge. Most TNB tests measure only accuracy, whereas most CNB tests measure both accuracy and speed. This is potentially important, as recent studies have shown that deviations from normal brain developmental trajectories are associated with significant deviations in performance for speed, but not accuracy [38]. For some domains (for example, attention), improved overall performance is reflected by better accuracy as well as faster speed. In contrast, in tasks that require more complex problem-solving (for example, PVRT and PMAT), we may see divergence in performance for accuracy and speed. Therefore, different analytic approaches may be required to interpret TNB and CNB performances across various domains. For example, for some analyses we plan to analyze accuracy separately, but with associated adjustments for speed.

### Imaging assessments

Our primary approach for analysis of sMRI, fMRI, and ASL MRI data in the NiCK study is to compare group differences in pre-specified ROIs, based on anatomic partitioning into structural/functional brain regions.

Using these pre-specified ROIs, we can explore specific hypotheses – for example, ROIs encompassing the prefrontal cortex and frontotemporal areas would be hypothesized to be affected by CKD, given literature describing deficits in executive function in this population [3]. We can therefore use the multimodal imaging approaches to compare characteristics of CKD and control subjects in these ROIs, using sMRI to detect volumetric differences, BOLD fMRI to test for differences in functional connectivity, and ASL MRI to compare regional CBF differences.

Whole brain approaches can also be used to explore the global effects of specific CKD comorbidities – for example, using ASL, we can examine the effects of hypertension and anemia on global cerebral blood flow (CBF), or CBF within gray matter and white matter compartments.

Additionally, *a priori* correlations with the neurocognitive data will be explored in an effort to identify underlying neurological processes for manifest neurocognitive dysfunction.

### Future directions

Although the primary analyses in NiCK will be hypothesis-driven, the nature of the imaging data collected also provides the opportunity to explore potential new connections and generate new hypotheses. For example, one hypothesis-free approach to analysis of the sMRI data uses voxel-based analysis [18,39] – this method evaluates the image data at its full resolution without *a priori* partitioning into anatomical ROIs. This unbiased discovery approach has the potential to yield new, previously unsuspected connections between regional brain abnormalities and CKD comorbidities.

A new machine learning methodology, the brain development index (BDI) [38], summarizes the multivariate pattern of structural brain development using a large sample of subjects ages 8–22, and can accurately delineate trajectories of brain development. Calculating and analyzing the BDI for NiCK subjects may potentially help us for identification of subtle developmental abnormalities related to CKD.

The long-term goal of the NiCK study is to develop a multi-parametric neuroimaging biomarker for patients with CKD. Using machine learning and pattern recognition methods, a classification system incorporating all the imaging measurements can be developed for individual patients. Multivariate pattern classification combining signals from sMRI, ASL and fMRI can be performed using the COMPARE (classification of morphological patterns using adaptive regional elements) [40] method. This combines signals from all imaging modalities to determine the set of brain regions and measurements that jointly offer the most distinctive set of measurements that characterizes the brain of CKD patients. In future studies, this sort of multi-parametric imaging index could be evaluated as a prognostic indicator to assess whether imaging findings can predict subsequent neurocognitive decline.

## Discussion

The NiCK Study represents the most comprehensive neurocognitive and neuroimaging phenotyping ever performed in children and youth with CKD. Our innovative approach of integrating multimodal MRI with neurocognitive and clinical data provides a unique opportunity to answer key questions in the field. These include understanding the independent neurologic impact of CKD, the neurologic effects of hypertension and other cardiovascular risk factors, and the consequences of CKD on neurodevelopment from childhood through young adulthood. Understanding these mechanisms could have great potential public health impact. First, this study could help to identify improved screening strategies to identify patients at high risk of adverse neurocognitive outcomes (for example, determining whether neuropsychological testing or neuroimaging should be incorporated into clinical care of youth with CKD). Second, neurocognitive impairment may affect adherence to the complex medical regimens that are routinely prescribed to patients with CKD. By improving our recognition of these deficits in our patients, we may be able to deliver health information in a more individualized and effective manner. Finally, by identifying mechanisms through which modifiable risk factors such as anemia and hypertension affect neurocognition, this study may help to guide targeted treatment strategies to prevent future cognitive decline.

## Abbreviations

ABPM: Ambulatory blood pressure monitoring
ASL MRI: Arterial spin labeled magnetic resonance perfusion imaging
BOLD fMRI: Blood oxygenation level dependent functional magnetic resonance imaging
BP: Blood pressure
CBF: Cerebral blood flow
CKD: Chronic kidney disease
CNB: Computerized neurocognitive battery
DBP: Diastolic blood pressure
eGFR: Estimated glomerular filtration rate
HR: Heart rate
MDRD: Modification of Diet in Renal Disease study
MRI: Magnetic resonance imaging
NiCK: Neurocognitive Assessment and Magnetic Resonance Imaging Analysis of Children and Young Adults with Chronic Kidney Disease Study
ROI: Region of interest
SBP: Systolic blood pressure
sMRI: Structural MRI
TNB: Traditional neurocognitive battery
